# Supplementary material for: Protein Levels of Pro-Inflammatory Cytokines and Chemokines as Biomarkers of Mycobacterium bovis Infection and BCG Vaccination in Cattle
Source: Pathogens. 2022 Jun 29;11(7):738. doi: 10.3390/pathogens11070738 (PMC9320177; doi:10.3390/pathogens11070738)
Supplement: Supplementary file 1 [file pathogens-11-00738-s001.zip › pathogens-1771645-supplementary.pdf]

Supplementary Table S1: Assay details of analytes evaluated in the study.

Analyte	Supplier	Coating Antibody Concentration	Composition of Coating Buffer	Composition of Blocking Buffer	Detection Antibody Concentration	Streptavidin HRP Dilution	Composition of Incubation/ dilution/reagent diluent buffers	Assay Range	Detection Limit / Sensitivity
IFNγ	Mabtech	2 µg/ml	PBS pH=7.4	0.05% Tween 20 and 0.1 % BSA	0.25 µg/ml	1 in 1000	0.05% Tween 20 and 0.1 % BSA	4-400 pg/ml	2.1 pg/ml
IL-2	Mabtech	2 µg/ml	PBS pH=7.4	0.05% Tween 20 and 0.1 % BSA	0.1 µg/ml	1 in 1000	0.05% Tween 20 and 0.1 % BSA	17-1700 pg/ml	50 pg/ml
IL-8	Mabtech	2 µg/ml	PBS pH=7.4	0.05% Tween 20 and 0.1 % BSA	0.1 µg/ml	1 in 1000	0.05% Tween 20 and 0.1 % BSA	8-800 pg/ml	17 pg/ml
IL-17A	Mabtech	2 µg/ml	PBS pH=7.4	0.05% Tween 20 and 0.1 % BSA	1 µg/ml	1 in 1000	0.05% Tween 20 and 0.1 % BSA	1-200 pg/ml	2 pg/ml
CXCL9	Biorad	1 µg/ml	Carbonate bicarbonate buffer pH=9.4	1 % BSA in PBS	0.2 µg/ml	1 in 400	0.05% Tween 20 in PBS	1-50 ng/ml	0.69 ng/ml
IP-10	Biorad	1 µg/ml	Carbonate bicarbonate buffer pH=9.4	1 % BSA in PBS	0.2 µg/ml	1 in 400	0.05% Tween 20 in PBS	1-50ng/ml	0.90 ng/ml
IL-1β	ThermoFisher	1: 100	Carbonate bicarbonate buffer pH=9.4	4 % BSA and 5% sucrose in D-PBS	1: 100	1 in 400	4% BSA in D-PBS	31-2000 pg/ml	26 pg/ml
IL-6	ThermoFisher	1:100	Carbonate bicarbonate buffer pH=9.4	4 % BSA and 5% sucrose in D-PBS	1: 100	1 in 400	4% BSA in D-PBS	78-5000 pg/ml	186 pg/ml
CCL4	KINGFISHER BIOTECH	1 µg/ml	D-PBS pH=7.4	4%BSA in D-PBS	0.1 µg/ml	1 in 400	4% BSA in D-PBS	1-25 ng/ml	0.03 ng/ml

PBS: Phosphate Buffer saline; D-PBS: Dulbecco's Phosphate Buffer Saline (Lonza); BSA: Bovine Serum Albumin (Sigma)

Supplementary Table S2: Levels of IFNγ, IL-2, IP-10, CXCL-9 and CCL4 in stimulated whole blood supernatants and serum

**Analyte**	**Group**	**Median Concentrations and Ranges (pg/ml)**		
		**Medium/Nil Stimulated Whole Blood Supernatants**	**PPDb Stimulated Whole Blood Supernatants**	**Serum**
IFNγ	Naïve cohort 1	0 (0,0)	242 (0,696)	0 (0,0)
	M. bovis challenged cohort	0 (0,7)	4000 (2962,5725)	0 (0,4000)
	Naïve cohort 2	0 (0,6)	292 (0,924)	0 (0,0)
	BCG Vaccinated cohort	0 (0,0)	214 (0,1109)	0 (0,141)
	BCG Vaccinated and *M. bovis* challenged cohort	0 (0,0)	835 (252,1664)	0 (0,0)
IL-2	Naïve cohort 1	230 (128,441)	401 (130,762)	376 (246,1591)
	M. bovis challenged cohort	568 (134,1504)	12703 (4571,20918)	293 (185,4250)
	Naïve cohort 2	36 (14,342)	67 (29,287)	26 (4,155)
	BCG Vaccinated cohort	252 (106,1249)	371 (264,1555)	0 (0,21)
	BCG Vaccinated and *M. bovis* challenged cohort	463 (258,3129)	2030 (883,10559)	122 (65,233)
IP-10	Naïve cohort 1	2975 (1389,14265)	3090 (1390,13636)	2975 (2123, 4326)
	M. bovis challenged cohort	11011 (0,44246)	69044 (26933,128126)	6778 (4448,74988)
	Naïve cohort 2	8132 (0,19516)	28073 (1662,12376)	9857 (3665,18426)
	BCG Vaccinated cohort	3803 (0,13574)	15040 (1537,41781)	8713 (6111,19038)
	BCG Vaccinated and *M. bovis* challenged cohort	2953 (1333,21740)	15989 (9108, 44460)	6998 (4299,7882)
CXCL-9	Naïve cohort 1	920 (296,7350)	770 (98,4597)	891.5 (500, 2235)
	M. bovis challenged cohort	6682 (778, 15773)	14480 (1702,17683)	13351 (2015,19135)
	Naïve cohort 2	110 (0,2642)	769 (0,5519)	896 (0,4189)
	BCG Vaccinated cohort	1772 (506,8100)	2152 (1013,8318)	1013 (0,2784)
	BCG Vaccinated and *M. bovis* challenged cohort	1241 (580,5912)	1740 (820,5598)	786 (639, 3174)
CCL4	Naïve cohort 1	510 (270,3900)	2265 (1000,5510)	140 (10,300)
	M. bovis challenged cohort	218 (90,19913)	27743 (16100,61254)	400 (20, 29760)
	Naïve cohort 2	0 (0,4254)	22982 (4987, 39569)	0 (0,50)
	BCG Vaccinated cohort	0 (0,456)	16789 (5732,30620)	0 (0, 73)
	BCG Vaccinated and *M. bovis* challenged cohort	315 (60,12650)	18570 (10290, 34300)	30 (20,80)

Supplementary Table S3: Levels of IL-8, IL-17A, IL-1β and IL-6 in stimulated whole blood supernatants and serum

Analyte	Group	Median Concentrations and Ranges (pg/ml)		
		Medium/Nil Stimulated Whole Blood Supernatants	PPDb Stimulated Whole Blood Supernatants	Serum
IL-8	Naïve cohort 1	612 (348,1644)	2013 (1472,2435)	476 (154,1051)
	M. bovis challenged cohort	790 (152, 8909)	2270 (983,6277)	670 (0,2939)
IL-17A	Naïve cohort 1	0 (0,0)	0 (0,3.1)	0 (0,0)
	M. bovis challenged cohort	0 (0,0)	125 (0,706)	0 (0,1222)
IL-1β	Naïve cohort 1	0 (0,61)	182 (40,629)	67 (51, 157)
	M. bovis challenged cohort	141 (4,1233)	583 (121,11701)	29 (0, 963)
IL-6	Naïve cohort 1	145 (0,3946)	936 (504,4145)	137 (0,2116)
	M. bovis challenged cohort	0 (0,13050)	1556 (118, 12348)	0 (0, 2860)

Supplementary Table S4: Potential of multibiomarker analysis to discriminate between animals that were *M. bovis* challenged and naïve animals.

(a) Analysis by comparing levels in bovine tuberculin (PPDb) stimulated whole blood supernatants					
**Animal Groups**	**NUM Score Cut-off**	**Sensitivity (95% CI)**	**Specificity (95% CI)**	**AUC**	**p-value**
Naïve vs. *M. bovis* challenged	>3	100% (70.09 to 100.0)	100% (80.64 to 100.0)	1	<0.001
(b) Analysis by comparing serum concentrations					
**Animal Groups**	**Cut-off**	**Sensitivity (95% CI)**	**Specificity**	**AUC**	**p-value**
Naïve vs. *M. bovis* challenged	> 3	88.89% (56.50 to 99.43)	100% (80.64 to 100.0)	0.9792	<0.001

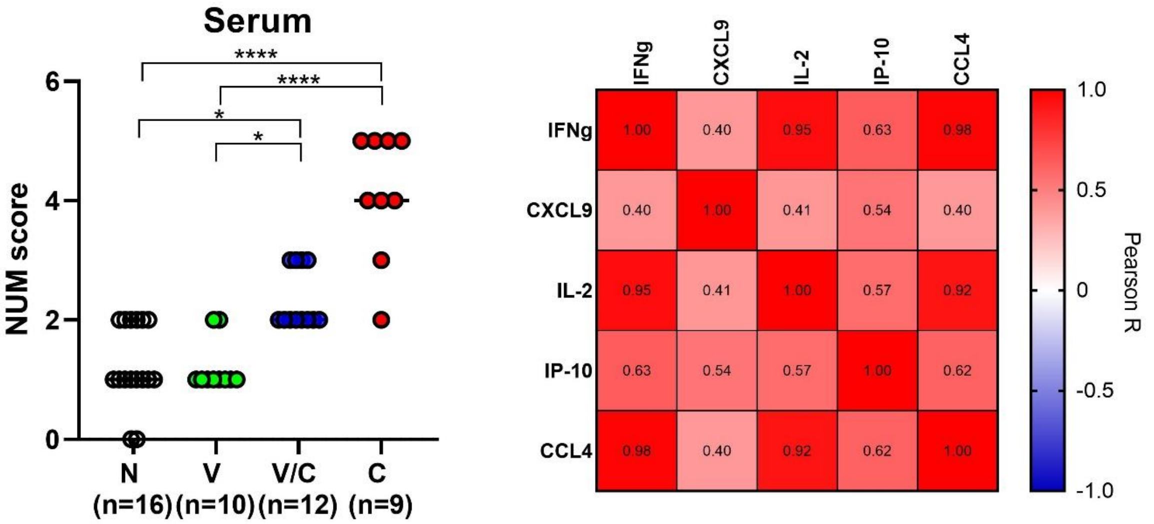

Supplementary Figure S1: NUM score, indicating the number of positive host proteins per animal calculated for serum samples (cut-off values determined via Youden's index), can be used to discriminate groups of naïve (N), BCG vaccinated (V) and *M. bovis* challenged animals with (V/C) or without (C) prior BCG vaccination (left). Heat-map showing correlation among concentrations of the evaluated host proteins (right) * $p < 0.05$, **** $p < 0.0001$.
